# Subcortical atrophy in frontotemporal dementia and Alzheimer’s disease: Significance for the differential diagnosis and correlations with clinical manifestations

**DOI:** 10.1590/S1980-57642009DN20400009

**Published:** 2008

**Authors:** Renata Teles Vieira, Leonardo Caixeta

**Affiliations:** 1 MSc. Behavioral and Cognitive Neurology Unit, Hospital das Clínicas, Federal University of Goiás, Goiânia (GO), Brazil.; 2 M.D, Ph.D. Associate Professor of Neuroscience, Federal University of Goiás (UFG). Coordinator, Cognitive and Behavioral Neurology Unit, Hospital das Clínicas-UFG.

**Keywords:** frontotemporal dementia, Alzheimer’s disease, structural neuroimaging, subcortical atrophy

## Abstract

Cerebral subcortical atrophy occurs in both Alzheimer’s disease (AD) and frontotemporal dementia (FTD) but its significance for clinical manifestations and differential diagnosis between these common types of dementia has not been extensively investigated.

**Objectives:**

To compare the severity of cerebral subcortical atrophy in FTD and AD and to analyze the correlations between cerebral subcortical atrophy and demographics and clinical characteristics.

**Methods:**

Twenty three patients with FTD and 21 with AD formed the sample, which comprised 22 men and 22 women, aged 33 to 89, with mean age (±SD) of 68.52±12.08 years, with schooling ranging from 1 to 20 years, with a mean (±SD) of 7.35±5.54 years, and disease duration with a mean (±SD) of 3.66±3.44 years. The degree of cerebral subcortical atrophy was measured indirectly with a linear measurement of subcortical atrophy, the Bifrontal Index (BFI), using magnetic resonance imaging. We evaluated cognition, activities of daily living and dementia severity with the Mini-Mental State Examination, Functional Activities Questionnaire and the Clinical Dementia Rating, respectively.

**Results:**

There was no significant difference (p>0.05) in BFI between FTD and AD. The severity of cognitive deficits (for both FTD and AD groups) and level of daily living activities (only for AD group) were correlated with BFI.

**Conclusions:**

A linear measurement of cerebral subcortical atrophy did not differentiate AD from FTD in this sample. Cognitive function (in both FTD and AD groups) and capacity for independent living (only in AD group) were inversely correlated with cerebral subcortical atrophy.

## Abstract (pt)

Atrofia subcortical cerebral ocorre na doença de Alzheimer e na demência frontotemporal (DFT) mas sua importância para as manifestações clínicas e para o diagnóstico diferencial não foram amplamente investigadas.

**Objetivos:**

Comparar a gravidade da atrofia subcortical cerebral na DA e na DFT e analisar as correlações entre atrofia subcortical cerebral e características demográficas e clínicas.

**Métodos:**

Vinte e três pacientes com diagnóstico de DFT e 21 com DA formaram a amostra que foi constituída por 22 homens e 22 mulheres, com idades entre 33 e 89 anos, idade média (±DP) de 68,52 (±12,08) anos, escolaridade variando de 1 a 20 anos, média de 7,35 (±5,54) e duração da doença com média de 3,66 (±3,44). O grau de atrofia subcortical foi avaliado indiretamente com uma medida linear de atrofia subcortical, o índice bifrontal (IBF) com o emprego de imagem por ressonância magnetica. A cognição, atividades de vida diária e gravidade da demência foram avaliadas com o Mini-Exame do Estado Mental, Questionário de Atividades Funcionais e Escore Clínico de Demência, respectivamente.

**Resultados:**

O IBF não foi diferente entre os grupos com AD e DFT (p>0.05). A gravidade do transtorno cognitivo (tanto para DA como DFT) e as atividades de vida diária (apenas para DA) correlacionaram-se com o IBF.

**Conclusões:**

Uma medida linear de atrofia subcortical não foi diferente entre pacientes com DA e DFT nesta amostra. A cognição (na DA e na DFT) e a capacidade de vida independente (apenas na DA) correlacionaram-se inversamente com a atrofia subcortical cerebral.

Diagnosis in life, of Alzheimer’s disease (AD) and Frontotemporal dementia (FTD) is made on clinical grounds, but currently used criteria are burdened with considerable subjective judgments,^1,2^ and yield an overall accuracy of 81% to 88% in AD cases.^3^ Given the high prevalence of both diseases and the increasing treatment options,^4^ simple and sensitive quantitative indicators of both forms of dementia in its early stages would represent valuable clinical tools. Measures of hippocampal atrophy have proven the most sensitive way of differentiating mild to moderate Alzheimer’s disease from non-demented elderly. Of these measures, the width of the temporal horn yields the highest sensitivity, predicting the disease in 73% of cases with 95% specificity.^5^

Differentiation between FTD and AD on neuroimaging, however, remains a great challenge, especially in the clinical setting.^6-11^

Cerebral atrophy occurs in almost all types of dementia and is characterized by a loss of global cerebral volume that can be indirectly observed by ventricular and cerebral sulcal enlargement.^12^ Sensitive imaging providing linear and volumetric measures of atrophy rates have been proposed to track this decline.^13-17^ Generally these measures are larger in patients with dementia than in healthy elderly.^18^

In this study, we aimed to better understand the relationship between the severity of cerebral subcortical atrophy and the type of dementia (FTD and AD), as well as to explore the relationship of age, duration and aggravation of dementia, educational level, daily living activities and cognition, with cerebral subcortical atrophy. Finally we test the usefulness of a linear measure of atrophy in differentiating AD from FTD.

## Methods

### Participants

A total of 44 participants diagnosed with dementia were recruited from the Clinicas Hospital at the Federal University of Goiás Medical School (FM-UFG), Brazil. There were no gender or ethnic restrictions. The study involved 22 men and 22 women, aged 33 to 89 years, with mean age (±SD) of 68.52±12.08 years, with schooling ranging from 1 to 20 years, with mean (±SD) of 7.35±5.54 years and disease duration with a mean (±SD) of 3.66±3.44 years.

The clinical diagnoses were reached by an experienced psychiatrist/neurologist (LC) based on patient history, neuroimaging results and neuropsychological tests. Diagnosis of dementia was based on the criteria of the Diagnostic and Statistical Manual Mental Disorders, Fourth Edition (DSM-IV).^20^

Etiology of dementia included patients with Alzheimer’s disease (n=21) and Frontotemporal Dementia (n=23). Diagnosis of FTD was based on Neary et al. criteria^21^ while the diagnosis of probable AD was based on the National Institute of Neurological Disorders and Communicative Disorders and Stroke-Alzheimer´s Disease and Related Disorders Association (NINCDS-ADRDA) criteria.^22^

Prior to carrying out this research, approval by the local research ethics committee was obtained (protocol number: 006/05). All subjects who agreed to participate signed a written informed consent.

### Instruments and procedures

#### Bifrontal index-BFI

**Figure 1:**
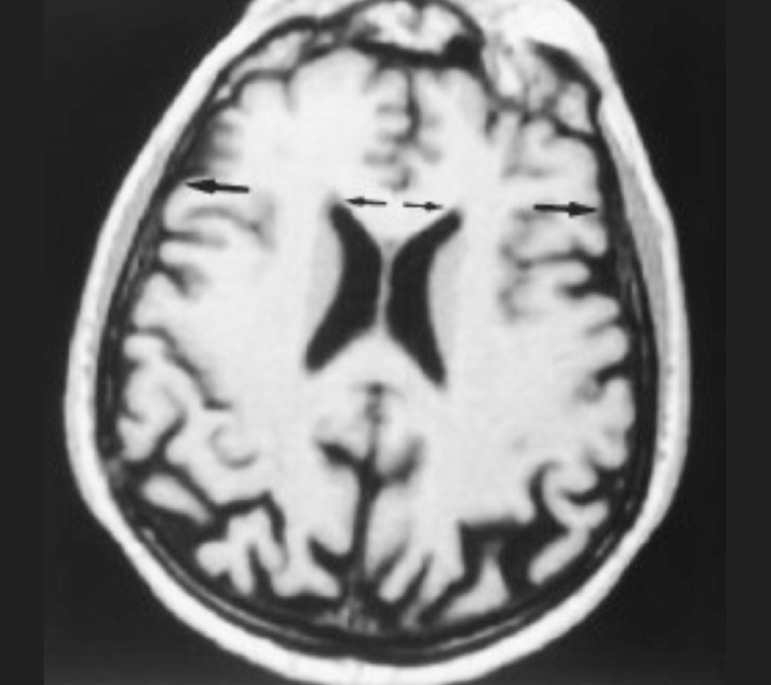
Axial MR image showing the width between the frontal horns of the lateral ventricles (smaller arrows) and the cranial width (larger arrows) – Bifrontal Index.

Magnetic resonance was performed on a 1.5–T MRI unit with a quadrature head coil. T1-weighted sequences were analyzed for this study. From the axial slice of structural neuroimaging (Magnetic Resonance Imaging), the BFI was measured on a plane parallel to the temporal lobe plane at the level of the maximum width between the tips of the frontal horns of the lateral ventricles, and defined as the ratio of this value to the diameter of the inner skull table at the same level. The resulting ratio was then multiplied by 100 and expressed as a percentage (Figure 1).^15,16,23,24^ A graded caliper with a 0.1 mm scale was used for this linear measurement on film copy.

The dilatation of the frontal horns of the lateral ventricle is one of the earliest changes seen in cerebral atrophy, while the BFI is a more reliable and practical linear measurement to predict early cerebral atrophy.^25^

***Clinical Dementia Rating (CDR)*** – Dementia severity was determined by total on the Clinical Dementia Rating Scale. The CDR assesses cognitive function in six domains: memory, orientation judgment and problem solving, community affairs and personal care. Based on six scores, a global CDR score is assigned in which CDR 0 indicates no dementia, CDR 0.5 indicates very mild dementia, CDR 1 indicates mild dementia, CDR 2 indicates moderate dementia, and CDR 3 indicates severe dementia.^19^

***Mini-Mental State Examination (MMSE)*** – All patients completed the MMSE at baseline, which was administered to determine cognitive function.^26^

***Pfeffer Functional Activities Questionnaire (FAQ)*** – Caregivers of dementia patients completed this questionnaire. It is a good instrument for assessing functional status, and includes ten questions on Activities of Daily Living (ADL).^27^

All 44 individuals were assessed using the BFI, CDR, MMSE and FAQ. Thus, duration of dementia and education level (in years) were also examined and served as inputs for the survival analysis. The neurological examination was performed during the same period as the clinical imaging. The patients were divided into two groups: one with FTD (*n*=23) and the other with AD (*n*=21). The BFI was compared in both groups for all analyzed variables.

#### Statistical analysis

We conducted all statistical analysis using the SPSS 12.0 software for Windows. The Mann-Whitney test (U) was performed to compare mean rates of variation between the two patient groups. The analyzed variables were: age, duration of dementia, MMSE scores, Functional Scale of Pfeffer’s scores, level of education in years, Clinical Dementia Rating Scale and BFI rate. We established the confidence interval as 95% for the statistical tests.

The Spearman Coefficient (rs) was used to obtain the correlation p and to verify the correlations between mean rates of brain atrophy (measured by BFI) and all other variables. The Spearman’s Coefficient was the non-parametric alternative when the data was not Gaussian and linear.

## Results

Table 1 shows the means (including standard deviation and confidence interval) of all the clinical features along with BFI for AD and FTD groups. Both patient groups were closely matched for age, duration of dementia, MMSE scores, Pfeffer Functional Activities Questionnaire (FAQ) scores and educational level. There was no significant difference in BFI between groups.

**Table 1:** Comparison of subcortical atrophy, demographic factors, disease severity and the duration of symptoms in patients with Alzheimer's disease and frontotemporal dementia.

	Patients with Alzheimer's disease (n=21)			Patients with frontotemporal dementia (n=23)		U	Z	p*
	M±SD	CI 95%		M±SD	CI 95%			
Age, y	73.52±7.94	69.90|-|77.14		63.95±13.47	58.12|-|69.78	598	-1.136	0.310^†^
Dementia duration,y	2.84±2.21	1.83|-|3.85		4.41±4.18	2.60|-|6.22	697	-0.245	0.376^†^
MMSE score	13.19±7.41	9.81|-|16.56		13.82±9.39	9.76|-|17.88	576	-1.178	0.298^†^
FAQ	22.00±10.34	17.28|-|26.71		20.04±10.45	15.52|-|24.56	818	-0.034	0.816^†^
Education, y	7.00±5.71	4.40|-|9.59		7.67±5.48	5.30|-|10.04	688	-1.29	0.358^†^
BFI	35.05±5.01	32.76 |-| 37.33		34.90±5.33	32.6 |-|37.21	556	-0.394	0.742^†^

* Significance on Mann-Whitney Test (U);

† No significant difference between groups p>0.05; MMSE, Mini-Mental State Examination; BFI, Bifrontal Index; EPSs, Extrapyramidal Signs; FAQ, Pfeffer-Functional Activities Questionnaire; M, Mean; SD, Standard Deviation; CI, Confidence interval; Z, standard normal deviation.

In the FTD group, only the MMSE score showed a strong correlation with BFI (Table 2). The AD group also showed a significant correlation between MMSE score and BFI, but weaker than that observed in the FTD group. There was a significant correlation (p<0.05) between BFI and Pfeffer Functional Activities Questionnaire (FAQ) scores in the AD group only.

**Table 2:** Correlation of Bifrontal Index Rate with demographic factors, disease severity and the duration of symptoms in the two groups.

	BFI				
	Patients with Alzheimer's disease			Patients with frontotemporal dementia	
	*Spearman's* correlation *(rs)*	*p* value		*Spearman's *correlation *(rs)*	*p* value
Age, y	0.282	0.216^‡^		0.214	0.326^‡^
Dementia duration,y	0.029	0.902^‡^		0.079	0.722^‡^
MMSE score	-0.491	0.024*		-0.647	0.001^†^
FAQ	0.495	0.023*		0.375	0.078^‡^
Education, y	-0.246	0.282^‡^		0.068	0.759^‡^
CDR	0.315	0.164^‡^		0.395	0.062^‡^

* Denotes p value of <0.05;

† Denotes p value of <0.001;

‡ Differences of modalities not significant (p>0.05); MMSE, Mini-Mental State Examination; CDR, Clinical Dementia Rating; BFI, Bifrontal Index; EPSs, Extrapyramidal Signs; FAQ, Pfeffer-Functional Activities Questionnaire

Age, duration of dementia and educational level were not correlated with BFI in either patient group (*p*<0.05). Other correlations were also not significant.

## Discussion

Indirect measures of subcortical atrophy, such as the BFI, Bicaudate Index and Ventricle-Brain ratio have been reported by many researchers to evaluate structural brain damage in patients with dementia. Both linear and volumetric measurements are probably more reliable than those made postmortem when ventricles are usually smaller than the same ventricles before death.^13-16,29,30^

AD and FTD can be difficult to differentiate clinically because of overlapping symptoms. Distinguishing the two dementias based on volumetric measurements of brain atrophy with MRI has been only partially successful.^9^ Our study did not demonstrate BFI differences between AD and FTD groups.

Age was not correlated with rates of BFI in either group across all analyses performed. This finding is consistent with the results reported by Brinkman et al.^33^ in the study of quantitative indexes of computed tomography in 28 patients with Alzheimer’s dementia and 30 elderly persons. Nevertheless, other authors^34^ have shown that age-related increases in BFI most probably reflect losses in adjacent brain structures including the caudate nuclei in normal aging.

Concerning the analysis of cognitive performance, measured by the MMSE, there was a negative correlation with BFI in both patient groups, mainly in the FTD group (p<0.001). This finding is in line with previous reports in the literature that have shown distinct types of cerebral changes predicting impaired performance on specific cognitive tests.^35-37^ Soderlund et al.^35^ also observed that subcortical atrophy estimated by means of ventricular enlargement were associated with cognitive deficits. Nevertheless, the measures used in the cited study were the BFI, the Caudate Ventricular Index and Occipital Ventricular Index. The average of the three indexes was used to calculate a global score. Furthermore, the 1254 participants had an MMSE score above 24 and were non-demented individuals.

A small number of studies have focused attention on the relationship between activities of daily living and linear brain measures in dementia patients, but only in Vascular Dementia and normal aging.^35,38^ Activities of daily living performance decreased with increased subcortical atrophy only for the AD group. Perhaps, one explanation for this fact is that FTD patients present a reduced capacity to perform daily tasks from the early stages of disease (a difference from AD),^39^ when BFI values still remain low.

We found no correlation between duration of symptoms and the linear measurement of subcortical atrophy. This may be expected because the extent of dementia is only an estimate. To our knowledge, no previous study has reported the association involving duration of dementia and subcortical atrophy measured by BFI.

We have also demonstrated that subcortical atrophy is not correlated with educational level. This could possibly be explained by the fact that participants had a large discrepancy in terms of years of education. Clinical pathological studies are necessary to clarify the association between subcortical atrophy and progression of dementia.

Studies including only one brain variable can be misleading because their putative association may be due to a correlated brain change while cerebral atrophy is an indirect measure of pathological processes occurring on a cellular level. In addition, the BFI is a non-specific finding which can result from brain injury or degeneration and which occurs normally in ageing, although many disease processes result in distinctive patterns of atrophy due to differential involvement of specific areas of the brain.

In conclusion, a linear measurement of subcortical atrophy such as BFI probably is not useful for providing a differential diagnosis between AD and FTD. Furthermore, cognitive function (in both FTD and AD groups) and capacity for independent living (only in AD group) decreased with increased subcortical atrophy. Our findings also revealed that age, duration of dementia and educational level do not significantly correlate with degree of cerebral atrophy.
